# Regulation of emotions in project-based collaborative learning: an empirical study in academic English classrooms

**DOI:** 10.3389/fpsyg.2024.1368196

**Published:** 2024-06-18

**Authors:** Lu Huang, Ruiying Wang, Jinlong Han

**Affiliations:** ^1^School of Information Technology in Education, South China Normal University, Guangzhou, China; ^2^School of Foreign Languages, South China University of Technology, Guangzhou, China; ^3^Faculty of College English Teaching, South China Business College Guangdong University of Foreign Studies, Guangzhou, China; ^4^Faculty of English Language and Culture, Guangdong University of Foreign Studies, Guangzhou, China

**Keywords:** PBCL, social emotion, self-regulation, co-regulation, socially-shared regulation

## Abstract

In spite of the increasing popularity of project-based collaborative learning (PBCL) as a pedagogy, real successful collaboration cannot always be achieved due to the cognitive, motivational and social emotional challenges students encounter during collaboration. Recognizing the challenges and developing regulation strategies to cope with the challenges at both individual and group level is essential for successful collaboration. In the last decades, a growing interest has been developed around socially shared regulation of emotions and how it is interwoven with self-regulation and co-regulation. However, capturing the process of students’ emotional challenges and regulations in a long and dynamic project proves difficult and there remains a paucity of evidence on how co-regulation and socially-shared regulation co-occur with learners’ cognitive and emotional progress in project-based collaborative learning. The purpose of the present study is to investigate and identify what kind of social emotional challenges students encountered during PBCL and how they regulate themselves and the groups in order to finish the projects. A quasi-experimental research design was adopted in an academic English classroom, with thirty-eight students self-reporting their challenges and regulations three times after finishing each of the projects. The results of qualitative analysis plus a case study of two groups indicate that students encounter a variety of social emotional challenges and employed different levels of co-regulation and socially shared regulation in addition to self-regulation, leading to varying collaboration results and experiences. The findings of the study offer insights into the emotional regulation in PBCL and shed light for future design of pedagogical interventions aiming at supporting socially shared regulation.

## 1 Introduction

With the advent of the era of knowledge economy and the development of artificial intelligence, the traditional way of teaching and learning has become increasingly difficult to meet the needs of future society. Cultivating students’ 21st century skills has become a consensus and important trend of education today and learning has been moving from a purely individual and externally programmed endeavor (i.e., planned and executed with the aid of a teacher) to learning in and with groups (Li et al., 2022). Collaborative learning, with its potential benefits of promoting higher-order thinking, communication, and leadership skills, has been advocated in almost all levels of education. In project-based learning (PBL) tasks, students need to go through a series of processes such as finding problems, collecting and analyzing data, communicating with each other, reflecting, etc., which can lead to deep learning and better learning effects on the one hand, and contribute to developing critical thinking, communication skills, teamwork spirit, problem solving and other critical skills on the other hand (Kim and Lim, 2018). Therefore, project-based collaborative learning (PBCL) is increasingly used as an innovative educational approach in a large variety of courses in higher education (Lin, 2018). Introducing PBCL into college academic English classrooms has also been found to not only positively influence the language skills and academic development of English learners, but also promote students’ higher-order thinking skills and academic identity construction, stimulate students’ independent learning, and cultivate students’ communication skills and team spirit (Li and Wang, 2018).

However, collaborative learning tasks are quite demanding for students as multiple individuals are required to share responsibility for a common goal, and learners will inevitably encounter cognitive, motivational, and emotional challenges (Järvelä et al., 2015). Project-based collaborative learning, with its complex ill-structured learning task, gives rise to even more challenges than traditional well-structured learning tasks (Volet et al., 2009). Engaging in PBCL, learners need to constantly negotiate and defend themselves, step out of their comfort zones, and change their initial thoughts and perspectives in an effort to seek a sense of identity and belonging, all of which may evoke more socio-emotional conflicts (Näykki et al., n.d.). Unresolved challenges and conflicts can lead to negative emotions, frustration, and even anger, weaken the process and results of collaborative learning, and even make it impossible for group projects to continue (Hämäläinen, 2012).

In order to resolve the challenges in collaborations, students need to engage themselves in regulation (Greene, 2018). Regulation is “intentional, goal-oriented meta-cognitive behavior through which learners monitor and control cognitive, affective, motivational, and behavioral aspects to achieve more ideal learning” (Boekaerts, 2011). Early regulation models mainly focused on the self-regulated learning (SRL) of individual learners and social context only played a mediating role in affecting cognition and the achievement of personal goals (Boekaerts et al., 2000). In the past two decades, more and more researchers from computer-supported collaborative learning (CSCL) moved their attention from individual regulation to social forms of regulation. They expand the SRL theory of educational psychology and stress that students in collaborative learning need to engage themselves in co-regulation and socially-shared regulation. By negotiating and constructing a shared regulation through deliberate and strategic planning, monitoring, controlling, and evaluation of cognition, motivation, emotions, and behaviors of a group, socially shared regulated learning (SSRL) has been recognized as an essential aspect of collaborative learning success (Panadero and Järvelä, 2015).

Nevertheless, it is a challenge to capture, measure and understand SSRL in authentic classrooms due to the highly interactive and dynamic nature of SSRL in collaboration. Following tradition of self-regulation, previous SSRL focused more on metacognitive strategies group members use to regulate their collective activity and relatively little attention was given to regulation of emotion and motivation. Therefore, little is known about what socio-emotional challenges group members encounter and how SRL, CoRL, and SSRL activities interweave with learners’ cognitive and emotional progress in collaborative learning (Nguyen et al., 2023). To fill the gap, more empirical research is needed regarding the regulation of emotion and how classroom teachers can design adaptive support and guide to promote students’ regulation in complex social learning situations in PBCL. Students’ experiences and attitude in collaborative learning is a “black box” for most teachers. Teachers lack sufficient understanding of the challenges students experience during collaboration, especially those related to emotional challenges, resulting in far insufficient guidance and support in collaboration tasks. This study aims to explore and identify the socio-emotional challenges encountered by students in PBCL and how group members employ strategies and construct regulation at both individual level and group level to ensure the smooth progress and accomplishment of the project.

## 2 Literature review

The concept and definition of regulation in the field of psychology can be traced back to the concept of “metacognition,” which refers to an individual’s knowledge about his or her own cognition and regulation of cognitive processes (Lavell, 1979). Researchers then further proposed the concept of “self-regulation” and pointed out that metacognition consists of two parts: learning-related knowledge that helps learners monitor and self-regulation mechanisms focusing on monitoring learning, including planning, identifying problems, modifying strategies, monitoring activities, evaluating and reviewing, etc. (Baker, 1984). The research on self-regulation in the field of educational psychology evolved and developed with the rise of constructivist learning theory in the 1980s. Today’s widely accepted definition of “self-regulated learning” (SRL) has gradually been expanded to include not only learners’ cognitive and metacognitive behaviors, but also motivation and emotion.

### 2.1 Social forms of regulation in collaborative learning

**Figure 1:**
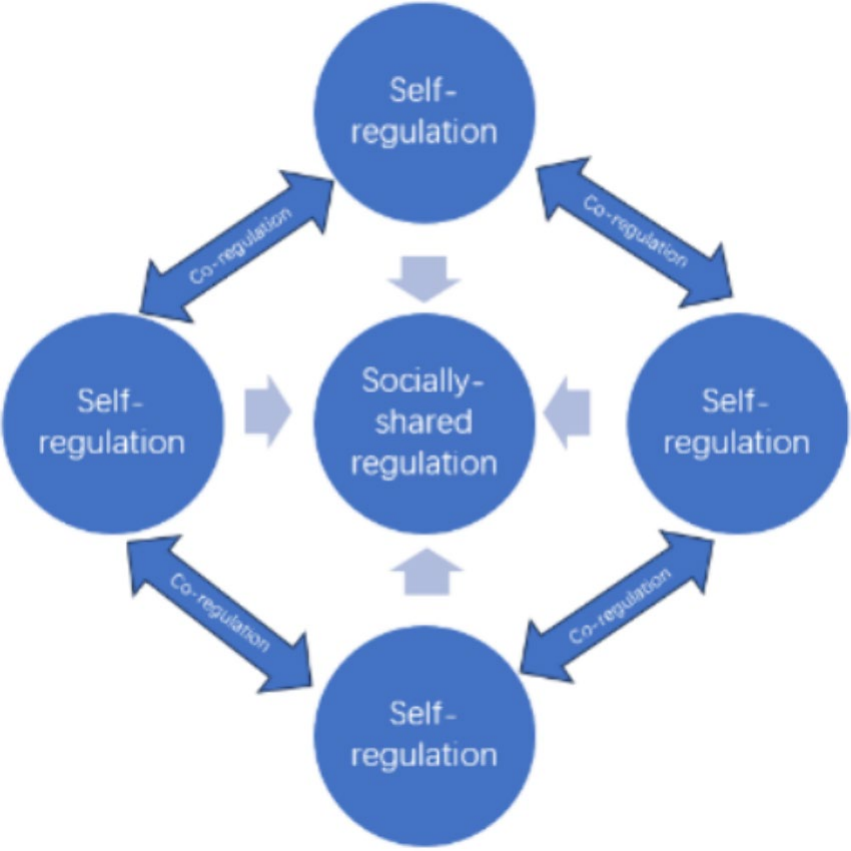
Three types of regulation in a collaborative group.

Self-regulation is defined as a learner’s ability to set and manage their own learning goals, monitor their performance, instruct themselves and reflect on their performance (Hadwin and Oshige, 2011). SRL is a core concept in educational psychology, giving rise to multiple models and a large amount of research based on these models over the past few decades (Panadero, 2017). These SRL models, from a social cognitive perspective, treats regulation as an individual process and focuses on the self-regulation of individual students even though they acknowledge the importance of social context in collaborative learning. The new model proposed by Hadwin et al. systematically defines and distinguishes three different forms of regulation in collaborative learning, namely, self-regulated learning (SRL), co-regulated learning (CoRL), and socially-shared regulated learning (SSRL) (Zimmerman, 1989). The model complements the traditional models of self-regulation from a social perspective, pointing out that students need to engage themselves in the three types of regulatory processes of different natures at the same time (Figure 1).

SRL as the strategic planning, execution, reflection, and adaptation learners make based on their personal interpretation of task and strategic knowledge is a process that occurs at all stages of learning (Järvelä et al., 2016). CoRL occurs when an individual’s regulated activities are directed, supported, modified, or constrained by other group members. SSRL occurs when a group regulates as a collective, such as by building and maintaining interdependent or collectively co-constructed regulatory processes, beliefs, and knowledge to achieve common outcomes. Among these three forms of regulation, self-regulation is well established and has been confirmed by many studies for the past decades. As for the two forms of social regulation, different theoretical perspectives have different focus. Researchers based on Vygotsky’s sociocultural theory emphasize the importance of co-regulation and believe that social interaction with more capable peers can promote the development of self-regulation. Research based on social construction theory, however, pays more attention to socially-shared regulation, believing that successful learners in collaboration should involve themselves in two processes: shared knowledge building and shared regulation. This socially-shared regulation in which multiple members jointly regulate their behaviors, motivations, and emotions in a synchronized and effective manner is the key to successful collaboration (Schunk and Zimmerman, 2008).

### 2.2 Socio-emotional challenges in PBCL

Emotional challenges and even conflicts are a natural part of human interaction and thus also of collaborative learning (Näykki et al., 2014). Compared with socio-cognitive conflict which can be positively related to team outcomes by elaborating different viewpoints through the conflict, socio-emotional conflict is regarded as negatively related to group cohesion, commitment, satisfaction and performance (Van den Bossche et al., 2006). Emotional challenges can emerge at different stages of collaboration for various reasons. Causes of challenges found in collaborative learning include individual differences, such as conflicting goals, different priorities or different working and communication styles. Conflicts in the collaboration process such as team members’ different levels of commitment and different ways of solving problems can also bring about challenges.

Regardless of the origin of the challenges, addressing and responding to the emotional challenges is critical to engaging all members in effective collaboration and ultimately ensuring that the group’s goals are achieved (Järvenoja et al., 2019). Emotional regulation is an individual’s capacity to understand others’ emotions, as well as the ability to modify the emotional experience when it is interfering with group goals and social interaction. (Boekaerts, 2011) While earlier researchers in individual learning context focused mainly on self-regulation, more and more studies found that effective self-regulated learners should be able to understand their own and others’ emotional reactions and strategically monitor and regulate emotion in the social environment that may interfere with the learning process (Nguyen et al., 2023). The field of CSCL has also gradually built a theoretical framework of socio-emotional regulation, establishing the importance of group socio-emotional regulation for successful collaborative learning (Kreijns et al., 2003).

Emerging emotions are shaped by the different task, cognitive, motivational, and interpersonal factors among which the students are situated and in pedagogical approaches such as PBCL, socio-emotional challenges can be heightened (Lajoie et al., 2015). In PBCL, students are usually given an ill-structured task, which involves collaboration, negotiation, and often cognitive dissonance, thus require more effort, persistence, and sustained motivation than traditional, well-structured tasks. Success of PBCL, therefore, is highly dependent on the learners’ socio-emotional regulation, which involves group processes invoked to address the emotions of the group (e.g., shared frustration over a negative group grade), resolve group challenges, or sustain positive interactions between group members. (Järvelä et al., 2016)

### 2.3 Socio-emotional regulations

Currently there are two lines of research studying socio-emotional regulation strategies in collaborative learning. Some researchers focus on the importance of social interactions during collaboration, believing that involving students in non-task-related interactions is necessary to develop positive relationships by prompting group members to understand each other and become a healthy learning community (Järvenoja and Järvelä, 2009). Good social interaction allows group members to gain a sense of relatedness, team cohesion, trust and mutual respect, making socially-shared regulation possible. Researchers found that some students use strategies of social reinforcement and task structuring to ensure quality of group interactions in collaboration (Andriessen et al., 2013).

Another line of research focuses on the social emotions triggered by various challenging situations in collaboration and the regulation strategies students adopted to cope with them. In the collaborative learning process, both the emergence of emotional conflicts and the regulation process usually go unnoticed, and it is not easy to accurately capture students’ social emotions in collaboration in a timely manner. Researchers can use students’ self-report after collaboration as well as technology-enhanced multimodal data during collaboration to reveal trigger events and capture challenges and social regulation. A considerable number of research focuses on how individuals attempt to regulate their own emotion and have linked SRL positively to academic achievement (Dent and Koenka, 2015). Others focus on social forms of regulation and found that groups engaging in more SSRL processes tend to engage in higher quality collaboration and enact deeper learning strategies (Ucan and Webb, 2015). Both CoRL and SSRL are regarded as being essential to productive collaborative learning as they can lead to sustained engagement as well as increased interaction and better communication among group members. (Su et al., 2018) In an exploratory study to determine what socio-emotional regulation strategies students utilized in a project-based learning environment, researchers found that students would target their emotions by using behavioral, interpersonal, cognitive, motivational, and a combination of cognitive and motivational strategies. Most of the strategies were used at SRL, CoRL and SSRL levels, but some were observed only at the group level (Lobczowski et al., 2021).

In spite of the increasing number of studies supporting positive impact of different forms of regulation in collaborative learning, more pedagogical evidence is still needed regarding socio-emotions that develop in social learning environments and whether and how students engage themselves in emotion regulation. Currently, a gap remains in the literature regarding emotion regulation forms and strategies used by students in project-based collaborative learning environments (Lobczowski et al., 2021). The present study aims to identify socio-emotional challenges encountered by students in academic English classroom and how they employ various forms of regulation in PBCL. Specific research questions this study tries to address are the following: (1) What kind of socio-emotional challenges do different groups of students experience in a project-based collaborative learning environment? (2) How do students regulate their emotions in coping with challenging situations and how do different forms of regulation interweave and interact in authentic collaboration? Our hypothesis is that different groups of students might encounter different challenging events or situations that may inhibit collaboration in conducting complex projects collaboratively. In such situations where emotions negatively impact learning and collaboration, SRL is not sufficient and students need to either regulate other students (CoRL) or negotiate and collectively regulate collectively (SSRL) in order to achieve personal goals and ensure the progress of collaboration.

## 3 Methods

### 3.1 Participants and research design

**Figure 2:**
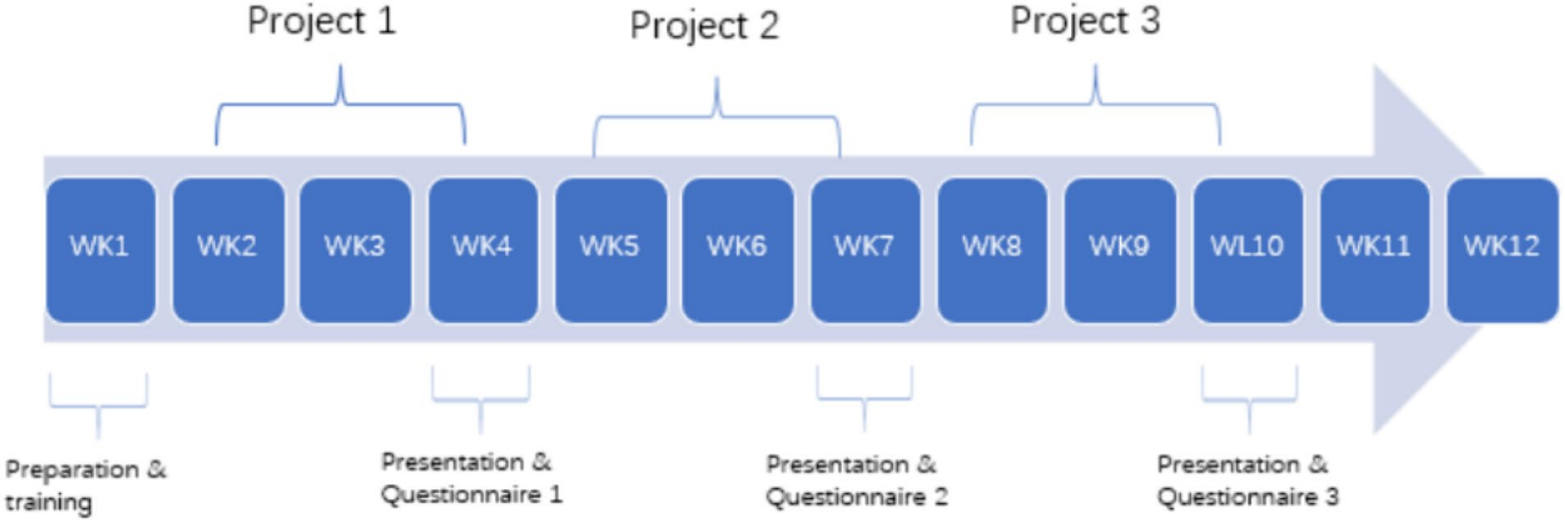
Pedagogical design and teaching schedule.

Participants of the present study were 38 (*N* = 38) first-year students, 16 female and 22 males students, majoring in software engineering from a university of technology in China and the experiment was conducted in a mandatory academic English class. English proficiency levels of all students are rated advanced as all students took the placement exam and were placed in A class. The teaching goal of this course is to improve students’ academic English proficiency, enabling them to engage in academic research, participate in academic conferences, and write academic papers in academic English. All students need to finish three projects collaboratively during 13 weeks within the same group and give an oral report of a group presentation as well as a written report. Students consented to participate were randomly assigned to nine groups of 4–5 people without considering their previous collaboration experiences and strategies. Topics of projects included shared bicycles, live streaming, social media, comparison of mobile operating systems, etc. Each project lasts for three weeks, and the process is led by students in a self-directed learning environment without teacher intervention. Most groups communicate through a combination of asynchronous discussions in WeChat groups, online meetings, and face-to-face synchronous discussions. The teacher and students from other groups will evaluate the project performance during the presentation (Figure 2).

### 3.2 Instrument

Regulation in collaboration is not only more complex, but also more difficult to measure. The research tradition of SRL regard regulation strategies as a kind of abilities that learners have when completing learning tasks and therefore mainly rely on questionnaires to measure learners’ ability to regulate their own cognition, motivation, and behavior. However, this measuring method assuming regulation as a relatively stable ability is difficult to capture the complex social forms of regulation involved in collaborative learning process. In the field of CSCL, many studies advocate capturing dynamic regulation by analyzing learner interactions with the help of technological tools and environment. The main limitation of this method is that both data Research Topic and analysis are extremely challenging and time-consuming, making it difficult to use in large-scale research and often limited to case studies. In addition, this method is difficult to investigate the complex social challenges students may experience over time because not all challenges experienced by learners can be observed and recorded.

To identify the real time socio-emotional challenges students experience and their efforts to regulate emotions at individual and group level during collaborative learning, Järvenoja et al. designed an Adaptive Tool for Emotional Regulation (AIRE) (Järvenoja et al., 2013). AIRE focuses on assessing learners’ experiences of socio-emotional challenges and how learners regulate emotions and motivation in different learning tasks and different situations, and is considered to mark a new stage in the development of socially shared regulation tools (Splichal, 2018). The AIRE tool is unique in that it seeks to capture the changing adaptive character of the entire regulation process by focusing on four interrelated components of student self-reported subjective experience.

The AIRE tool was used in the present study because the 13-week long project-based collaborative learning tasks is a longitudinal asynchronous experiment design and this tool is particularly suitable for repeatedly measuring students’ change of emotional challenges and corresponding regulation strategies gradually developing over time to cope with different situations in each collaborative learning task.

### 3.3 Data collection

Data for this study consisted of questionnaires adapted from the AIRE tool completed by all students shortly after each project presentation. The purpose, structure and filling method of the questionnaires were explained to the students in detail before they filled it out for the first time. All students filled out the same questionnaires with the same items after each project, and 102 valid questionnaires were recovered. In addition to using questionnaires for quantitative analysis, two focus groups were also selected for in-depth interviews. With the consent of the eight students in the two groups, further qualitative analysis was conducted on their questionnaires and interviews. The chat log of the group chat is also provided by the two groups to gain insight into their real time interaction.

The questionnaires consist of four parts, which not only include the challenges students experience in collaborative learning situations (part 2) and students’ emotional regulation of challenges at the individual and group levels (part 3), but also include group members’ personal goals (part 1) and goals achievement reflection on the situation (part 4). All the socio-emotional challenge scenarios and regulation types follow the original instrument of AIRE with only some minor change of wording to fit the academic English projects. The second part (Table 1) gives twelve socio-emotional challenges divided into five categories: personal priorities, work and communication, team work, collaboration, and external constraints. Students rated the challenges they encountered that triggered social–emotional regulation using a five-point Likert scale (0 = did not happen, 4 = big challenge). The questionnaires give specific examples of each challenge to help students to understand the specific challenges. For example, challenges regarding incompatible styles of working are that some students wanted to start right away while others wanted to plan first and start to work after that. After rating the challenges, students were asked to pick two out of 12 as their biggest challenges.

**Table 1:** Socio-emotional challenges categories and possible situations.

Categories	Possible challenging situations
Personal priorities	A. Our goals for the project were different
	B. We had different priorities
Work and communication	C. We seemed to have incompatible styles of working
	We seemed to have different styles of interacting.
	E. People in our group did not connect very well with one another
Team work	F. Some people were not fully committed to the group project
	G. People had very different standards of work
	H. Group members were not equal.
	I. Some people were easily distracted
Collaboration	J. Our ideas about what we should do were not the same
	K. We differed in our understanding of the concepts/task
External constraints	L. We had different personal life circumstances or family /study & work commitments

The third part (Table 2) aims to identify different forms of regulation by giving choices of 12 regulatory strategies used by students to control emotions and maintain motivation when faced with the two biggest socio-emotional challenges, including four self-regulation strategies, four co-regulation strategies and four socially-shared regulation strategies. Students were also asked to use a five-point Likert scale to rate the frequency with which specific coping strategies were used at both the individual and group levels (0 = did not happen, 4 = happen a lot).

**Table 2:** Self-regulation, co-regulation, and socially-shared regulation strategies reported.

Type of regulation	Example of strategies to cope with challenges
Self-regulation (SR)	I convince myself that it could actually be a good thing, because…
	I tried to act more flexible.
	I tried to understand that the others were not simply trying to be difficult but they had different goals.
	I tried to accept the situation and realize that some people were prepared to put in more work than others.
Co-regulation (CoR)	I told the others that we needed to accept that some people were prepared to put in more work than others.
	I told the others we needed to be more flexible in order to find a compromise/solution for the situation.
	I tried to explain to others that we needed to understand different goals.
	I tried to convince someone that the others were not simply trying to be difficult and we can solve the situation.
Socially-shared regulation (SSR)	We understood that we have to reconcile our goals closer to one another.
	We solve the situation by compromising to accommodate everyone’s goals.
	We decided that we had to work out the situation together in order to carry on working.
	We accepted that different members have different goals and we organized our working according to that.

In order to better understand the challenges encountered by students, the first part allows students to choose their most important personal goals in the project, while the fourth part allows students to reflect and evaluate whether their own goals have been achieved and whether the group has played a positive or negative role. To better adapted to Chinese students’ learning purpose for academic English projects, students were only given 8 options out of the 12 options in original instrument.

## 4 Results

### 4.1 Descriptive data and analysis

Based on the data collected in the second part of the 102 questionnaire responses, the frequencies of two biggest challenge types reported by students in each project and the sum of the frequency of the biggest challenge types in all three projects were calculated. A one-sample, chi-squared test was performed across the three tasks to test the distribution of different challenge types. Even though students worked in the same group, the situation was different in each task and the challenges were presumably different. Therefore, students’ responses were treated as independent, although the same students responded to the same questions after each project. Variations within the challenge types and a chi-squared test of the relationship between challenges and different projects/times are also presented to indicate changes in the challenges. For the answers in the third part, the means and standard deviations of the three regulation forms reported by the students were calculated. Mean values across challenge types and three different forms of task regulation were analyzed using one-way ANOVA tests.

The greatest challenges reported by students spanned the 12 challenges listed and the frequency varied across the three different projects (Table 3). In terms of the total frequency of the three projects, the most frequent type reported by students is teamwork (30.8%); followed by challenges related to work and communication (24.3%), and challenges related to the collaboration (20%). Relatively minor challenges are those related to personal priorities (16.8%), and challenges related to external constraints (8.1%). The chi-squared test confirmed that this distribution of the challenges did not occur by chance (Table 3).

**Table 3:** Frequencies and proportions of the different challenge types reported.

Challenge categories	Project 1		Project 2		Project 3		Total	
	f	%	f	%	f	%	f	%
Personal Priorities	14	21.5	11	17.5	6	10.5	31	16.8
Work & communication	16	24.6	16	25.4	13	22.8	45	24.3
Team work	20	30.8	18	28.6	19	33.3	57	30.8
Collaboration	10	15.4	12	19	15	26.4	37	20
External constraints	5	7.7	6	9.5	4	7	15	8.1
Total	65	100	63	100	57	100	185	100

A chi-squared analysis did not indicate a significant difference in the distribution of challenges between the tasks. However, the biggest challenges reported by students changed as the teams progressed from the first to the third project, with challenges related to the collaboration increasing (15.4, 19, and 26.4%, respectively), while challenges with work and communication fluctuating (24.6, 25.4 and 22.8% respectively). Challenges related to personal priorities changed considerably, ranging from 21.5% in the first project to 17.5% in the second and only 10.5% in the third. The frequency of challenges related to external constraints remained consistently lowest (7.7, 9.5, and 7%) from the first to the third project, respectively.

As for the regulation types, students reported all three different types of regulation: self-regulation, co-regulation, and shared regulation across all their projects. Regardless of the project or type of challenges, students reported both self-regulation and social forms of regulation. In general, self-regulation and socially- shared regulation appear more frequently than co-regulation (Table 4). The means of regulation did not differ across the different challenges and tasks when analyzed using a one-way ANOVA test.

**Table 4:** Means and standard deviations of self-regulation, co-regulation, and socially-shared regulation strategies reported.

Regulation	Projects							
	Project 1		Project 2		Project 3		Sum	
	M	SD	M	SD	M	SD	M	SD
Self-regulation	7	3.7	6.8	4.4	6.5	3.7	6.7	3.8
Co-regulation	5.2	3.1	4.5	2.8	3.7	3	4.4	3.1
Socially-shared regulation	7.2	4.3	7.5	4.9	8.1	4.9	7.6	4.7

### 4.2 Case studies

It is worth nothing that the frequency of challenges and shared regulation experienced by different individual within the same group can vary greatly. In order to better explain the consistency or differences in students’ shared regulation reports and gain an insight into the different dynamics between groups, the questionnaires of two different groups (Group C and Group F) after the end of project 2 were selected for detailed comparison. These two groups were selected because Group C had the best performance in group presentation, and all group members clearly stated that their collaboration was very pleasant. Group F, however, encountered a very big crisis during the project and even asked the teacher for help. Questionnaires from the two groups were selected for further qualitative analysis. Each group member’s detailed responses regarding personal goals, group accommodation, three forms of accommodation, goal achievement, and group evaluation were put into group files. All members in the two groups were also invited for an semi-structured in-depth interviews to provide more detailed descriptions of their questionnaires. In accordance with the questionnaire, the interview questions also focused on students’ perception of their collaboration and conflicts and challenges encountered by the groups, strategies taken to cope with challenges.

**Figure 3:**
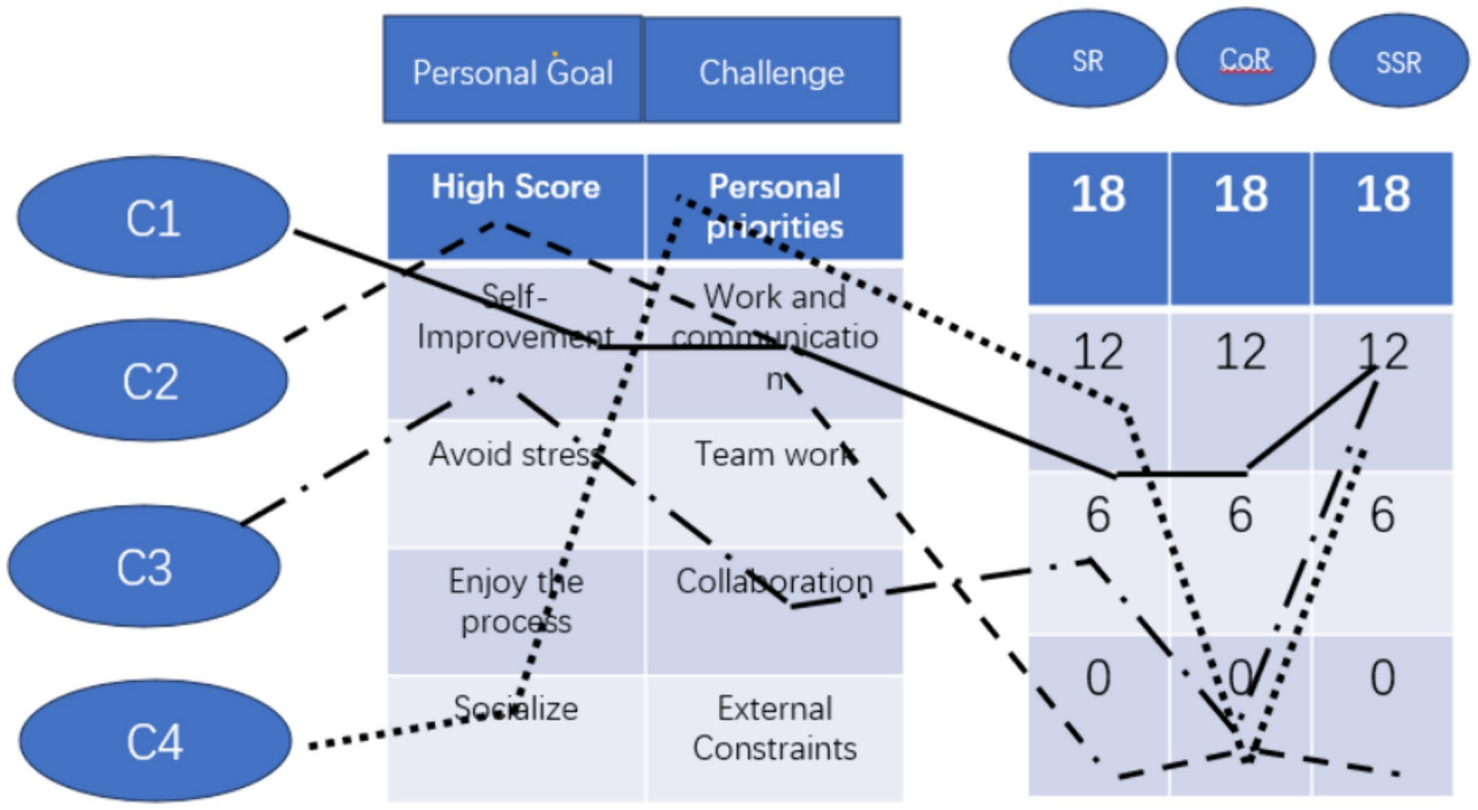
Profile of group C after project 2.

Group C (Figure 3) consists of three boys and one girl. According to their self-reports on the questionnaire, their personal goals are different, some aimed to improve their English but others pay more attention to socializing and learning from others. Most of their perceived socio-emotional challenges came from work and communication. C1 and C2 reported that the main challenge came from different communication styles, C3 and C4 reported that the challenge was different from personal standards for work, but even the biggest challenge they selected was 1 on the scale, which means very small challenge. Facing the challenge, except for C2 (C2 = 1), the other three members all reported more socially-shared regulation (C1 = 10 C3 = 9 C4 = 7), followed by self-regulation.

An interview with all four members reveals that they found no big socio-emotional challenges in their group and most challenges can be resolved by regulating themselves. They only need to activate socially-shared regulation on rare occasions when there were negative emotions. In accordance with the results of the questionnaire, the group members were able to act together in order to achieve their personal goals although interpretations of the challenging situation differed.

**Figure 4:**
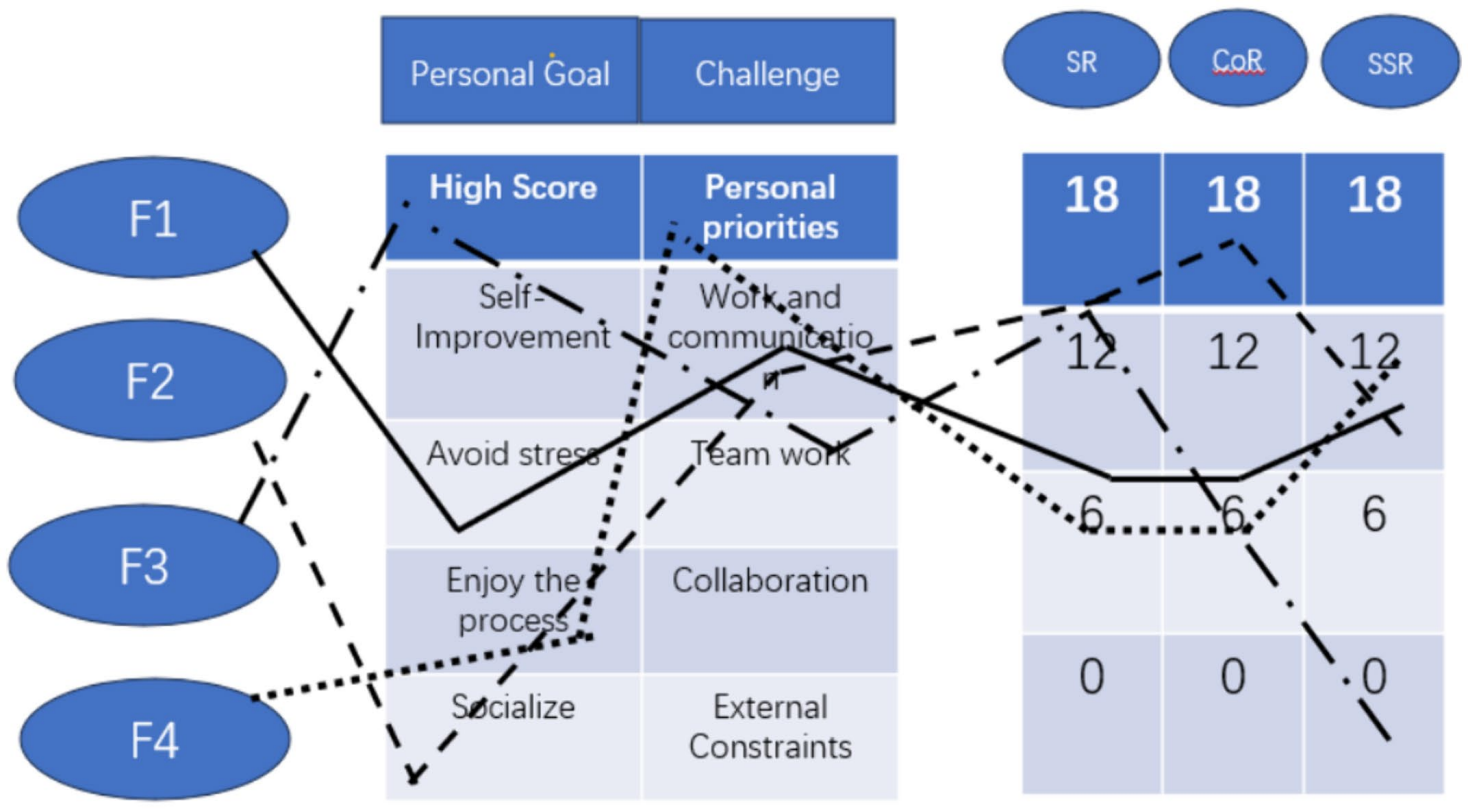
Profile of group F after project 2.

Group F (Figure 4) consists of two boys and two girls. According to their self-reports, the four students had considerable differences in their personal goals and experiences of socio-emotional challenges. The goal of F1 is to avoid stress and negative results; the goal of F2 is to learn from each other and socialize; the goal of F3 is to get high scores; and the goal of F4 is to enjoy the process. All four students reported a maximum level of challenge of 4 (big challenge), especially in the work and communication category. F3 particularly reported as many as 7 out of 12 socio-emotional challenges, and all rated 3 or 4. However, in the face of so many challenges, F3 chose to engage herself more in self-regulation (F3 = 12) instead of co-regulation with other students (F3 = 5) and socially-shared regulation (F3 = 2), while the other three group members all worked very hard to construct shared regulation (F1 = 9 F2 = 8 F4 = 11) within the group. F2 assumed more responsibilities than others--she not only applied more self-regulation (F2 = 12), but also worked very hard to regulate other team member (F2 = 15) in order to maintain the group cohesion and pushed the project to proceed normally as planned (Figure 4).

**Figure 5:**
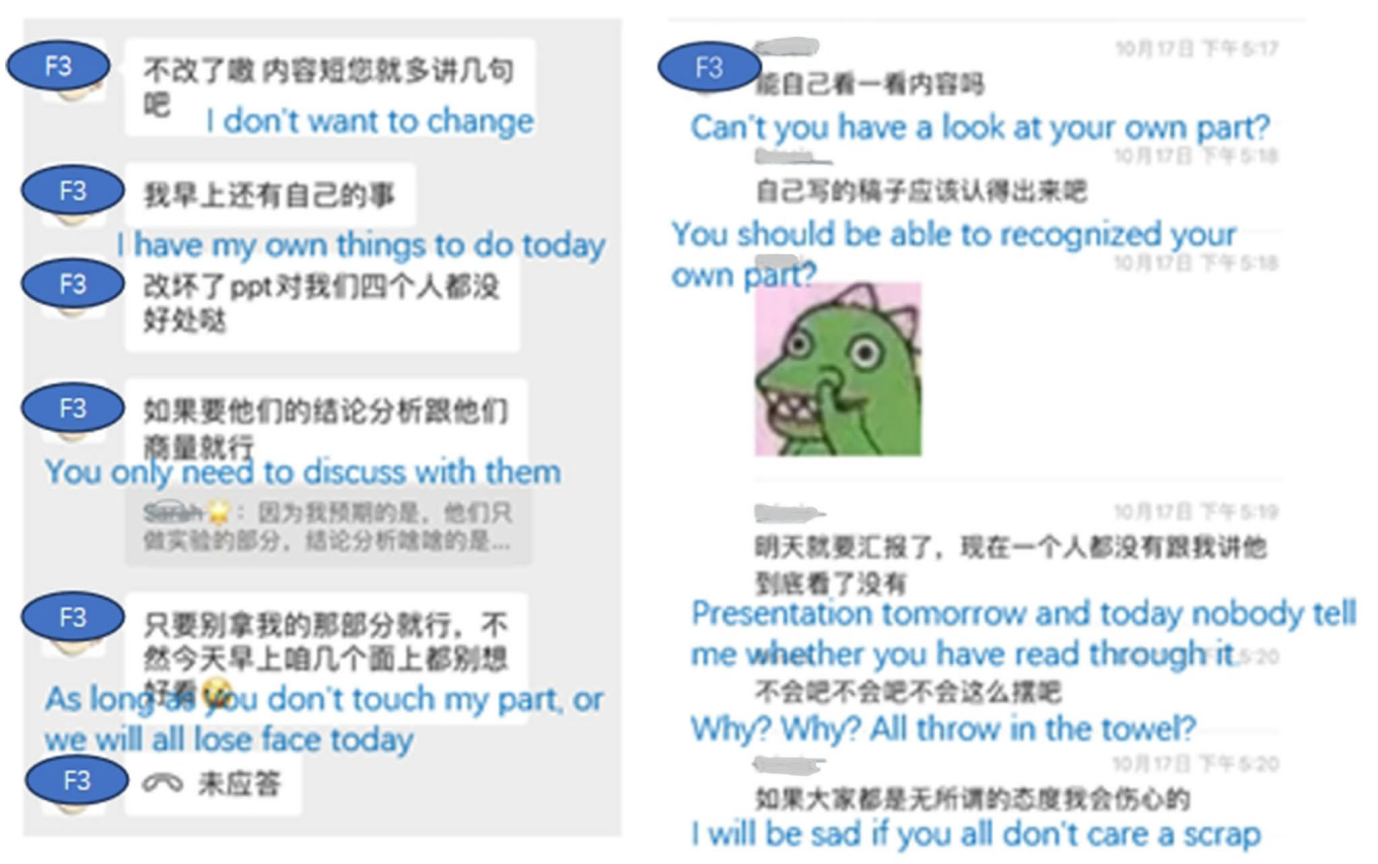
Overruling and undermining interaction by F3.

Chat log of group F revealed that both F2 and F3 did a lot of work for the group while F1 and F4 were not very active. But as the project progressed, a negative feeling gradually emerged in the group. Interpersonal dynamics were troubled even dysfunctional interactions occurs in group F. Analyzing the interaction of group F, the type of interaction that challenged the group’s collaboration include overruling and undermining interaction (see Figure 5) found in previous studies (Barron, 2003). The interview reveals that the major reason was due to the differences in individual goals and interpretations of the situation or “a lack of a common ground,” as mentioned by F3.

**Figure 6:**
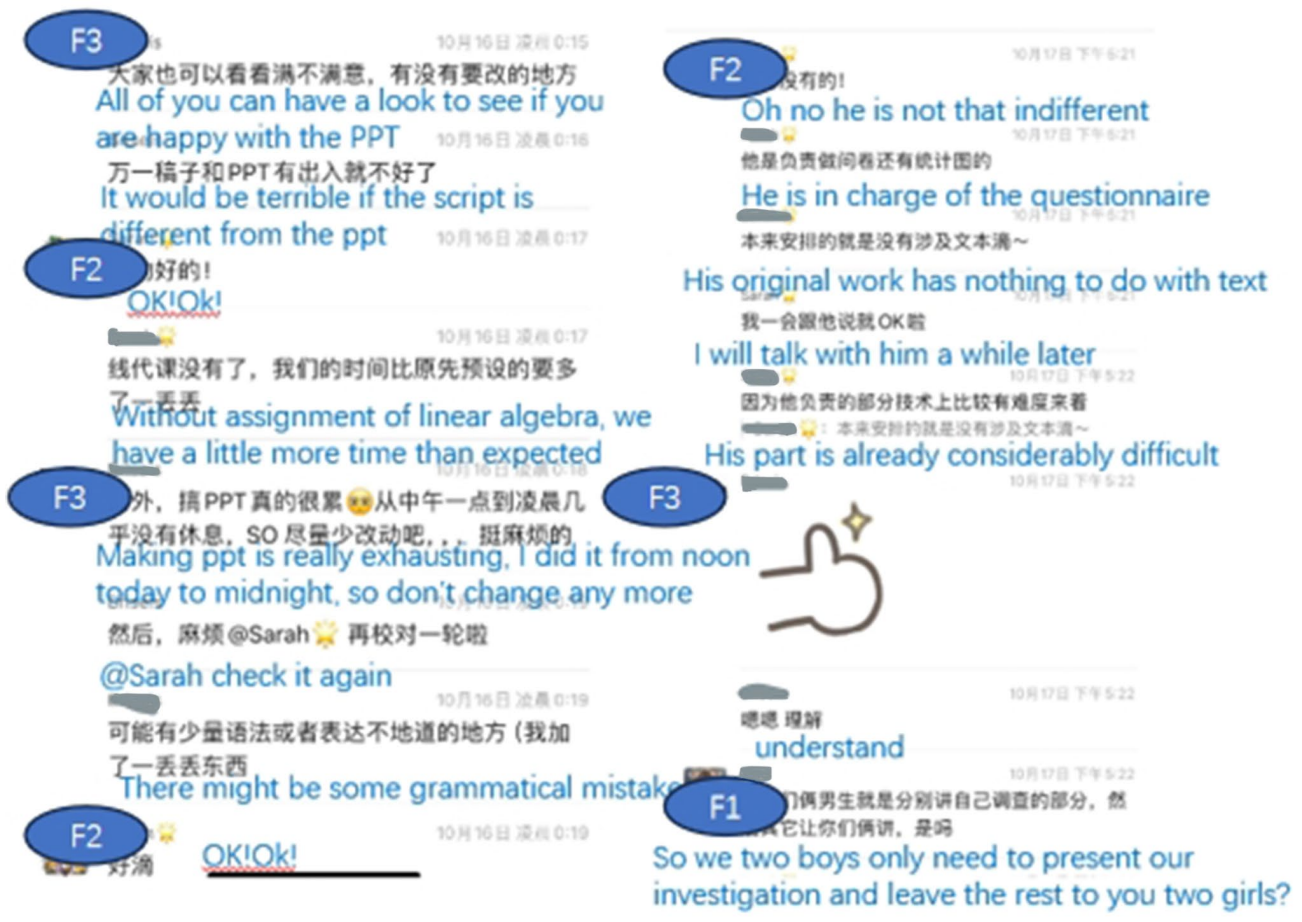
Examples of socio-regulation in group F.

As for the regulation of the emotion, the interview revealed that all four members tried what they could to regulate the big socio-emotional changes, especially F2 and F3. F2 was the only one who attempted to co-regulate the emotion of the group but the negative atmosphere made the various regulation strategies less effective. Faced with the increasing tension, F3 focused on proceeding with the task by using metacognitive regulation like dividing the jobs among the students (see Figure 6) instead of engaging herself in socio-emotional regulation, making it impossible for the whole group to reach a high level of shared-regulation.

## 5 Discussion

The present empirical study of project-based collaborative learning investigates the emotional challenges experienced by college students and their regulation strategies used to cope with the challenges during their PBCL in academic English classroom. The following are the conclusions.

### 5.1 Emotional challenges vary within groups and between projects

Consistent with the findings of some previous research, almost all students more or less experience a variety of socio-emotional challenges during the complex project-based learning process and the biggest emotional challenges centered around two sources, namely, differences in teamwork and challenges related to work and communication. Case study reveal that different groups can have totally different experiences--students in Group C experienced fewer challenges, and the degree was very low even if they did. In Group F, on the contrary, almost all student felt considerable challenges. One member (F3) felt seven out of the 12 challenges, including not committing enough by some team members, different work and communication styles, different attitude toward work, different standards, unequal relationships, lack of connection, etc. The challenges felt by other group members mainly come from the way of communication with F2.

Previous research has shown that both the pedagogical structure and the students’ increasing experience of one another may affect the nature of the challenges they encounter. In PBCL, ill-structured tasks resulted in more challenges regarding the different working and interacting styles of group members remain high in some groups from the first project to the last one. Overruling and Undermining interaction narrowed the participation possibilities of the group members (Barron, 2003) and this type of interaction was visible throughout the group F chat. Unlike findings of previous research which shows that with the gaining experience of project-based learning and increasing familiarity of team members, students’ perceived challenges can decrease steadily, the present study shows that real classroom situation is much more complicated. Emotions are socially constructed, but they are experienced personally (Näykki et al., 2014). Students’ experience and interpretation of socio-emotional challenges will affect their emotional experience and even if there is only one member who failed to optimize her strategic regulation when these challenging events occur, it could lead to dysfunctional groups as well as maladaptive individual and group learning performance.

### 5.2 Socially-shared regulation is crucial for successful PBCL

Most groups of the present study engaged in different forms of regulation when faced with challenges, including self-regulation, co-regulation, and socially-shared regulation, with socially-shared regulation being most frequent and co-regulation least frequent. Case comparison shed light on differences between groups, showing that the greater the perceived challenges, the more self-regulation strategies and socially shared regulation strategies were used. In the case of group F, F2 engaged in a lot of socially-shared regulation as well as self-regulation to make the group project proceed smoothly. Contrary to F2, F3 was faced with the most challenges, but only tried to solve the problem through self-regulation and failed to construct the socially-shared regulation with her teammates. It is worth noting that in Group C, there were also students like C2 who reported very little socially-shared regulation but did not prevent the whole group to construct socially-shared regulation. This might due to the fact that all members of group C experienced much less challenges than group F. From questionnaires and interviews, C2 was found to be the weakest student in the group and he was very dependent on other students in the group, contributing very little to the group. Unlike F2 who gave a lot of troubles to her group, C2 was a typical “free rider.”

Previous research has shown that when students encounter socio-emotional challenges, they may self-regulate or co-regulate, and students tend to engage in socially-shared regulation when they encounter social-cognitive challenges (Meyer and Turner, 2006). This is partly in line with the findings of the present study. The emotional challenges felt by students in Group C were very low and the main challenges came from the cognitive challenges related to the project. Therefore, students in the group did not need to co-regulate and engaged themselves mostly in socially-shared regulation. However, the findings of less successful groups like Group F are contrary to that of the previous study. For Even though all members in Group F perceived big emotional challenges, only F2 tried very hard to co-regulate but in vain. F3 only resorted to using metacognitive regulation, which made the situation even worse. The findings of the present study indicate that self-regulation of all members and socially-shared regulation of three members still could not prevent the groups from failing. Unlike previous research which emphasized critical role of socially-shared regulation (Frijda, 2005), co-regulation of emotion is also very important for coping with the socio-emotional challenges in collaboration.

## 6 Conclusions and implications for future research

The limitations of this study should be noted. Since it is a semi-experimental study conducted in a natural classroom setting, the sample size of the data was not large enough. As a diachronic study, the duration of the time was also not long enough. Future research on social–emotional regulation can be improved from the following aspects.

The present study is based on students’ self-report of socially-shared regulation, which might not be the real shared-regulation emerging in the collaboration process. It is suggested that more informative measures for revealing regulatory processes in collaborative learning is needed. The latest CSCL research emphasizes the use of multimodal data with the help of artificial intelligence to capture shared regulation in collaborative learning process in order to better monitor the real-time status of regulation in collaboration (Splichal, 2018).

Findings of this search also point out the need to support socio-emotional regulation in PBCL. Some researchers have proposed that CSCL tools can be used to support regulation in collaboration, as long as the supported goals move from the knowledge building process to the regulation process (Miller and Hadwin, 2015). The present study indicated that failure in regulating resulted in the failure of collaboration and groups need support and guidance to promote ideal social–emotional interactions (Järvelä et al., 2023). It is worth noting that repeated use of questionnaire tools such as AIRE enables students to understand their learning goals and enhance their awareness of regulation process through reflection and the tool in itself can serve as support of regulation.

Research on metacognition and self-regulation emphasizes the importance of the development of strategy, which gradually becomes more automatic and eventually transferable to new learning situations. Developing interventions that scaffold and support students’ skills of regulation would be a worthwhile future direction (Kim and Lim, 2018). Studies have shown that providing students with external scripts for reflection in project-based learning can effectively promote the formation of internal scripts for students’ socially-shared regulation (Splichal, 2018). As the project continues to progress, students will continue to adjust their regulation strategies (Kwon and Liu, 2014). The benefits of using project-based learning in academic English classrooms can go beyond language learning and prove to be valuable to coping with the emerging challenges in higher education.

## Data availability statement

The raw data supporting the conclusions of this article will be made available by the authors, without undue reservation.

## Ethics statement

The studies involving humans were approved by South China University of Technology Ethics Committee. The studies were conducted in accordance with the local legislation and institutional requirements. Written informed consent for participation in this study was provided by the participants' legal guardians/next of kin. Written informed consent was obtained from the individual(s) for the publication of any potentially identifiable images or data included in this article.

## Author contributions

LH: Writing – review & editing, Writing – original draft. RW: Writing – review & editing, Methodology, Data curation. JH: Writing – review & editing, Supervision, Methodology.
